# Effects of reduced sedentary time on resting, exercise and post-exercise blood pressure in inactive adults with metabolic syndrome – a six-month exploratory RCT

**DOI:** 10.1038/s41371-024-00894-6

**Published:** 2024-01-24

**Authors:** Jooa Norha, Tanja Sjöros, Taru Garthwaite, Saara Laine, Maria Saarenhovi, Petri Kallio, Kirsi Laitinen, Noora Houttu, Henri Vähä-Ypyä, Harri Sievänen, Eliisa Löyttyniemi, Tommi Vasankari, Juhani Knuuti, Kari K. Kalliokoski, Ilkka H. A. Heinonen

**Affiliations:** 1 Turku PET Centre, University of Turku and Turku University Hospital, Turku, Finland; 2 Department of Clinical Physiology and Nuclear Medicine, University of Turku and Turku University Hospital, Turku, Finland; 3 Paavo Nurmi Centre and Unit for Health and Physical Activity, University of Turku, Turku, Finland; 4 Institute of Biomedicine, University of Turku, Turku, Finland; 5 The UKK Institute for Health Promotion Research, Tampere, Finland; 6 Department of Biostatistics, University of Turku and Turku University Hospital, Turku, Finland; 7 Faculty of Medicine and Health Technology, Tampere University, Tampere, Finland

**Keywords:** Risk factors, Hypertension

## Abstract

Evidence on the long-term effects of reducing sedentary behaviour (SB) on blood pressure (BP) is scarce. Therefore, we performed a sub-analysis of the BP effects of a six-month intervention that aimed at reducing SB by 1 h/day and replacing it with non-exercise activities. Sixty-four physically inactive and sedentary adults with metabolic syndrome (58% female, 58 [SD 7] years, BP 143/88 [16/9] mmHg, SB 10 [1] h/day) were randomised into intervention (INT, *n* = 33) and control (CON, *n* = 31) groups. Resting BP and BP at each stage during and after a graded maximal bicycle ergometer test were measured before and after the intervention. SB, standing, moderate-to-vigorous physical activity (MVPA), and light physical activity (LPA) were measured in six-second intervals at baseline and during the whole six-month intervention using hip-worn accelerometers. The analyses were adjusted for BP medication status. The intervention resulted in a 40 min/day reduction in SB and concomitant 20 min/day increase in MVPA. Resting systolic BP was lower in the CON group before and after the intervention. No group x time interactions were observed in resting BP or BP during exercise at submaximal or maximal intensities, or during recovery. The changes in LPA and MVPA were inversely correlated with the changes in BP during light-to-moderate intensity exercise. An intervention that resulted in a 40 min/day reduction in SB for six months was not sufficient at influencing BP at rest, during or after exercise in adults with metabolic syndrome. However, successfully increasing LPA or MVPA might lower BP during light-to-moderate-intensity activities.

## Introduction

The global burden of arterial hypertension remains high with higher than 30% prevalence [1]. One-third of adults with hypertension additionally have metabolic syndrome, which further increases the risk for cardiovascular events [2]. In addition, adults spend most of their waking time (9.3 h/day, on average in Finland) in sedentary behaviour (SB) [3], which is defined as ≤1.5 metabolic equivalent (MET) sitting or reclining activities [4]. While the role of physical activity (PA) in hypertension prevention and treatment is well-established [5, 6], less is known about the effects of reducing SB on blood pressure (BP).

In observational studies, high self-reported SB is associated with slight increases in BP and an increased risk for hypertension in both children and adults [7]. Furthermore, longer duration of accelerometer-measured SB is associated with higher 24 h and nocturnal BP in adults [8]. Additionally, based on cross-sectional modelling, replacing SB with light PA (LPA) might result in a BP reduction [9].

Experimental studies on the effects of SB-reducing interventions on BP are scarce. Short-term studies suggest that breaking up prolonged SB reduces systolic (SBP) and diastolic BP (DBP) acutely by 4.4 mmHg and 2.4 mmHg, respectively [10]. Furthermore, workplace-based intervention studies focused on interrupting SB have shown a reduction in BP after a 13-week [11] or 12-month intervention period [12], with reductions ranging from 1 to 11.5 mmHg. However, these studies had some limitations regarding a lack of a control group and not measuring SB or PA during the intervention.

BP measurement during physical exertion can provide valuable information on vascular health even in normotensive individuals [13, 14]. Normally, SBP increases linearly, on average, by 4.9 mmHg/MET with increasing workload in healthy individuals [15]. After exercise is terminated, SBP starts to decline at a rate of roughly 10 mmHg/min [16]. However, a steeper increase in SBP and impaired BP recovery after maximal exercise are associated with an increased risk of all-cause mortality and myocardial infarction [15, 17]. During exercise testing, peak BP is most often used as a defining feature of an abnormal BP response even though no definitive consensus on the definition of an abnormal BP response exists [18].

In this six-month intervention study, we investigated the effects of reducing SB without adding intentional exercise training on resting and exercise BP in physically inactive adults with metabolic syndrome. We hypothesised that reducing SB without exercise training might yield improvements in BP in individuals with high baseline SB, physical inactivity, and cardiovascular risk factors (i.e., metabolic syndrome). Additionally, we assessed whether changes in PA of different intensities and body composition associate with changes in BP.

## Materials and methods

The data in the current study consists of secondary outcomes of a randomised controlled trial conducted at the Turku PET Centre, Turku, Finland, between April 2017 and March 2020 (pre-registered at Clinicaltrials.gov NCT03101228, 05/04/2017). The study was conducted according to the Declaration of Helsinki and the Ethics Committee of the Hospital District of Southwest Finland gave approval for the study (16/1801/2017). Participants gave their informed consent before entering the study.

### Participants

As reported previously [19], participants for this study were recruited from the community by newspaper and bulletin board advertisements. Inclusion criteria were self-reported physical inactivity (<120 min of weekly moderate-to-vigorous physical activity [MVPA]), high sedentary time (≥10 h or ≥60% of accelerometer wear time during screening), age 40–65 years, overweight or obesity (body mass index (BMI) 25–40 kg/m^2^), and metabolic syndrome [20]. Exclusion criteria included resting SBP ≥160 and/or DBP ≥100 mmHg, history of any cardiac disease, fasting blood glucose ≥7.0 mmol/l or diagnosed diabetes, abundant alcohol consumption (according to the Finnish guidelines: >12 or >23 units/week for women and men, respectively), the use of any tobacco products or narcotics, diagnosed depressive or bipolar disorder, previous exposure to ionising radiation, inability to understand written Finnish, and any condition that would be detrimental to the participant or the study procedures.

### Measurements

All of the measurements were performed at baseline and after the intervention period.

### Blood pressure

Resting brachial BP was measured seated with the arm supported at heart-level 2–3 times using a digital oscillometric sphygmomanometer (Apteq AE701f, Rossmax International Ltd, Taipei, Taiwan) after at least a 10-min seated rest, and an average of these readings was used. Due to the device used, the measurements were not performed unattended. The cuff size was chosen to fit the individual’s arm circumference. The participants were advised to take their BP medications as usual. The use of BP medications was self-reported by the participants.

The graded maximal bicycle ergometer test was executed as previously reported [21]. In brief, the test was started at 25 W and the load was increased by 25 W every three minutes until volitional fatigue, a medical reason for termination (e.g., SBP >260 mmHg), or refusal to continue. A recumbent bicycle ergometer (eBike EL Ergometer with Case v6.7, GE Medical Systems Inc., Milwaukee, WI, USA) was used. The test was considered as maximal when the respiratory exchange ratio was >1.0, a plateau in oxygen uptake was achieved or the heart rate reached ±10 beats/min of age-predicted maximum. Before the exercise test, resting BP was measured manually in a seated position. During the test, BP was measured manually after one minute on each workload. Finally, BP recovery was measured one and three minutes after exercise termination in a seated position. The rate of BP decline from the end of exercise to the recovery measurements at 1 and 3 min (i.e., BP_recovery_ – BP_last_) was used as the outcome. The BP examiner was blinded to the group allocation. However, it cannot be ascertained that the participants did not reveal their group allocation unprompted.

Exercise BP was calculated for individually determined intensities (i.e., 25%, 50%, 75%, and 100% of maximum power output). Additionally, we calculated maximal METs (1 MET = 3.5 ml O_2_ /kg/min) achieved during the exercise test. Then, we calculated the rate of SBP increase per 1 MET (SBP/MET-slope) using the formula (SBP_max_ – SBP_baseline_)/(MET_max_ – 1) as described by Hedman et al. [15].

### Accelerometry

As reported earlier in more detail [19], each participant’s PA and SB habits were measured using hip-worn triaxial accelerometers for an initial four-week screening period (UKK AM30, UKK Terveyspalvelut Oy, Tampere, Finland) and during the whole subsequent six-month intervention period (Movesense, Suunto, Vantaa, Finland). The accelerometry data was recorded in six-second epochs according to our standard procedures [22]. PA was divided into LPA and MVPA using the validated mean amplitude deviation method [22]. Furthermore, <1.5 MET activities were classified as either SB or standing using the validated angle for posture estimation method [23]. In addition to the absolute amount in h/day, PA and SB were also calculated as a proportion (%) of accelerometer wear time to account for individual differences in wear time. Additionally, the number of daily steps and breaks in SB were calculated [23]. Wear time of 10–19 h/day and a minimum of four days of accelerometry was considered valid.

### Anthropometry

Body mass, body fat percentage and fat free mass were measured using validated air displacement plethysmography (Bod Pod, COSMED USA Inc., Concord, CA, USA) after at least four hours of fasting. Height was measured using a wall-mounted stadiometer. BMI was then calculated using the standard formula body mass (kg) / height (m)^2^.

### Intervention

After screening, the participants fulfilling the inclusion criteria were randomised into the intervention (INT) or control (CON) group by a statistician using permuted block randomisation in a 1:1 ratio and block size 44. Randomisation was performed separately for men and women in SAS (version 9.4 for Windows) to ensure balanced groups.

The details of the six-month intervention have been published previously [19]. In short, the INT group was instructed to reduce SB by 1 h per day compared to the individual average during screening and concomitantly increase daily standing, LPA, or MVPA by a total of 1 h. The goals and ways of reducing SB were set and discussed individually. A maximum of 20 min/day was added to MVPA. Each participant received tips and support in meeting the goals in a one-hour individual counselling session. In the CON group, the daily PA and SB goals were set equal to the individual average during screening. Both groups were advised to maintain their usual physical exercise training habits. All participants were able to monitor their daily PA and SB on a mobile phone application (ExSed, www.exsed.com, UKK Terveyspalvelut Oy, Tampere, Finland) [24].

### Statistical methods

In the main analyses, each participant was analyzed in the group they were originally allocated to. The analyses were performed using linear mixed models for repeated measurements. The models included time as a within-factor variable and group and the interaction term group x time as the independent variables. The outcome of interest (i.e., BP) was the dependent variable. The analyses were adjusted for sex and BP medication status (yes/no). Furthermore, when analyzing BP during the exercise test, intensity (i.e., at rest before the exercise test, 25%, 50%, 75%, and 100% of maximal power output) was included as a second within-factor variable in addition to the aforementioned variables. Tukey-Kramer adjustment was used for multiple comparisons and a compound symmetry or unstructured covariance structure was used for time, choosing the appropriate one based on the Akaike information criterion. The normal distribution of the residuals was evaluated visually. Sample size was determined based on power calculations for the main outcome of the study (whole-body insulin sensitivity) [19].

Descriptive statistics are presented as mean (standard deviation [SD]), unless stated otherwise, and the intervention effects are presented as model-based least squares means (95% confidence interval [CI]). Statistical significance was set at *p* < 0.05 (two-tailed). The linear models were analyzed in SAS 9.4 (SAS Institute Inc., Cary, NC, USA) and baseline characteristics and correlations were calculated using IBM SPSS Statistics 28.0 (IBM, Armonk, NY, USA).

### Additional analyses

Additional exploratory analyses were performed because no intervention effects were observed in the main analyses. First, we divided the participants according to the measured mean change in SB during the whole intervention into a “less sedentary” group (SB reduction of ≥3 percentage points of accelerometer wear time; *n* = 34) and a “continuously sedentary” group (SB reduction <3 percentage points or an increase in SB; *n* = 30). Then, we divided the participants according to the change in total PA (MVPA + LPA) into a “more active” group (total PA increase of ≥3 percentage points of accelerometer wear time; *n* = 33) and a “less active” group (total PA increase <3 percentage points or a decrease in total PA; *n* = 31). Three percentage points of accelerometer wear time translates to about 27 min/day with 15 h/day wear time. Additionally, these cut-points resulted in relatively even-sized groups. Participants with missing accelerometer data (*n* = 8) were allocated according to the original randomisation in the exploratory analyses. The main linear mixed model analyses were replicated using these group divisions.

Second, we analyzed Pearson’s correlations between the changes (Δ) during the intervention period in the accelerometry, anthropometric (i.e., BMI and body fat percentage) and BP variables. Further, the correlation analyses between the changes in accelerometry and BP variables were repeated with Pearson’s partial correlation adjusting for the change in BMI. The correlation analyses were performed in the whole study group, regardless of group allocation.

## Results

In total, 263 volunteers were initially assessed for eligibility. After screening, 64 eligible participants were randomised into the INT (*n* = 33) or CON (*n* = 31) groups. A total of four participants discontinued the study (one in the intervention and three in the control group) (see Supplementary Fig. 1, CONSORT Flow diagram). Fifty-eight percent of the participants were female, mean age was 58 (SD 7) years, BMI was 31.3 (4.3) kg/m^2^ and resting SBP and DBP were 143 (16) and 88 (9) mmHg, respectively. Fifty-two percent of the participants reported using BP medication. Mean SB at baseline was 10 (1) h/day. Baseline characteristics of the participants are presented in Table 1.

**Table 1:** Baseline characteristics of the participants.

	Total (*n* = 64)	Intervention (*n* = 33)	Control (*n* = 31)
Female participants, *n* (%)	37 (58)	20 (61)	17 (55)
Age, years	58.3 (6.9)	59.3 (6.0)	57.2 (7.5)
Body mass, kg	93.2 (16.1)	92.4 (16.6)	94.1 (15.8)
BMI, kg/m^2^	31.6 (4.3)	31.5 (4.0)	31.7 (4.6)
Body fat, %	43.1 (7.9)	43.1 (8.0)	43.1 (8.0)
FFM, kg	52.9 (10.8)	52.6 (11.9)	53.2 (9.8)
BP medication, *n* (%)	33 (52)	16 (49)	17 (55)
Resting SBP, mmHg (SD; min–max)*	143 (16; 109–176)	146 (15; 112–176)	139 (16;109–169)
Resting DBP, mmHg (SD; min–max)*	88 (9; 70–112)	89 (8; 74–112)	88 (9; 70–104)
Maximal power output, W	130 (31)	128 (33)	132 (30)
Maximal MET	6.49 (1.33)	6.47 (1.44)	6.50 (1.24)
Maximal SBP, mmHg	216 (22)	218 (22)	214 (23)
Maximal DBP, mmHg	91 (12)	95 (12)	88 (12)
SBP change from rest to maximal, mmHg	79 (21)	79 (22)	79 (20)
DBP change from rest to maximal, mmHg	4 (11)	6 (10)	2 (12)
SBP/MET-slope, mmHg/MET	15 (4)	15 (5)	15 (3)
1 min recovery SBP, mmHg	−12 (18)	−11 (15)	−13 (20)
3 min recovery SBP, mmHg	−50 (21)	−48 (21)	−53 (21)
Accelerometry, days	26 (4)	26 (4)	26 (3)
Wear time, h/day	14.54 (0.97)	14.47 (0.96)	14.60 (1.00)
Sedentary behaviour, h/day	10.04 (1.01)	10.02 (0.92)	10.06 (1.11)
Standing, h/day	1.79 (0.59)	1.81 (0.61)	1.76 (0.57)
Light PA, h/day	1.74 (0.44)	1.67 (0.40)	1.81 (0.48)
Moderate-to-vigorous PA, h/day	0.97 (0.32)	0.96 (0.31)	0.97 (0.34)
Steps, n/day	5149 (1825)	5204 (1910)	5091 (1760)
Breaks in sedentary time, n/day	29 (8)	28 (8)	29 (8)

All data is presented as mean (standard deviation, SD) if not stated otherwise.

*BMI* body mass index, *FFM* fat free mass, *BP* blood pressure, *SBP* systolic blood pressure, *DBP* diastolic blood pressure, *MET* metabolic equivalent of task, *PA* physical activity.

*This is the baseline value which may be different from the screening value that was used to assess inclusion criteria.

### Intervention results

As reported earlier, SB decreased during the intervention in the INT group by 40 min/day and remained unchanged in the CON group [19]. MVPA increased in the INT group by 20 min/day whereas no significant changes in the CON group were observed [19]. Daily step count increased in both groups but more in the INT group (INT 3300 vs. CON 1600 steps/day) [19]. Standing time remained unchanged in both groups and LPA increased by 10 min/day in both groups with no between-group differences [19].

**Fig. 1:**
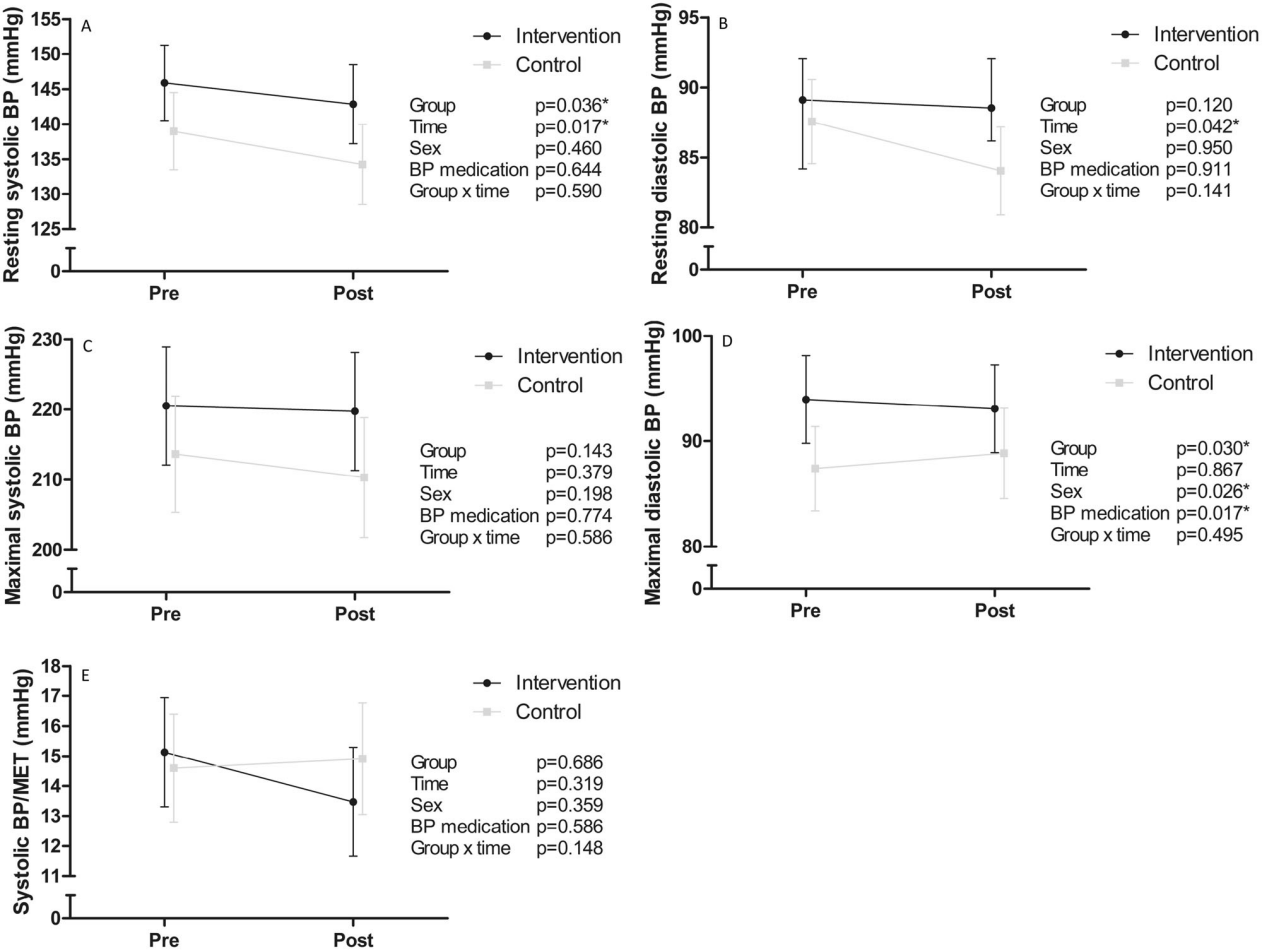
Intervention effects on blood pressure. Systolic (**A**) and diastolic (**B**) blood pressure at rest, maximal systolic (**C**) and diastolic (**D**) blood pressure during exercise testing, and systolic blood pressure increase per one metabolic equivalent of task during exercise testing (**E**) in the intervention (black dots) and control (grey squares) groups before (pre) and after (post) the intervention. Estimates are model-based least squares means and error bars represent 95% confidence intervals. BP blood pressure, MET metabolic equivalent of task. *p < 0.05.

**Fig. 2:**
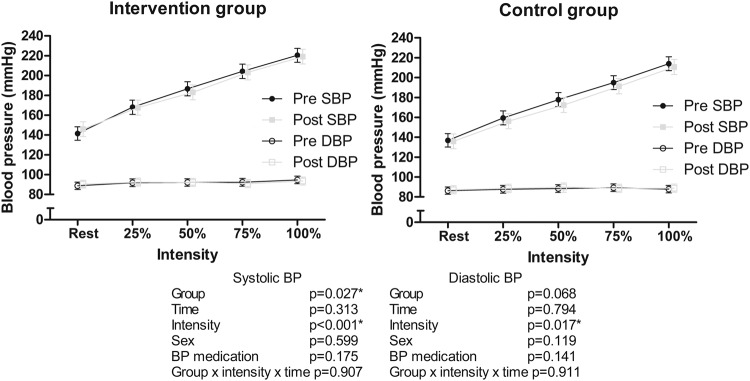
Systolic and diastolic blood pressure at rest before the exercise test, and at 25%, 50%, 75%, and 100% of maximal power output in the intervention (left panel) and control (right panel) groups before (black circles) and after (grey squares) the intervention. Estimates are model-based least squares means and error bars represent 95% confidence intervals. SBP systolic blood pressure, DBP diastolic blood pressure, BP blood pressure. *p < 0.05.

**Fig. 3:**
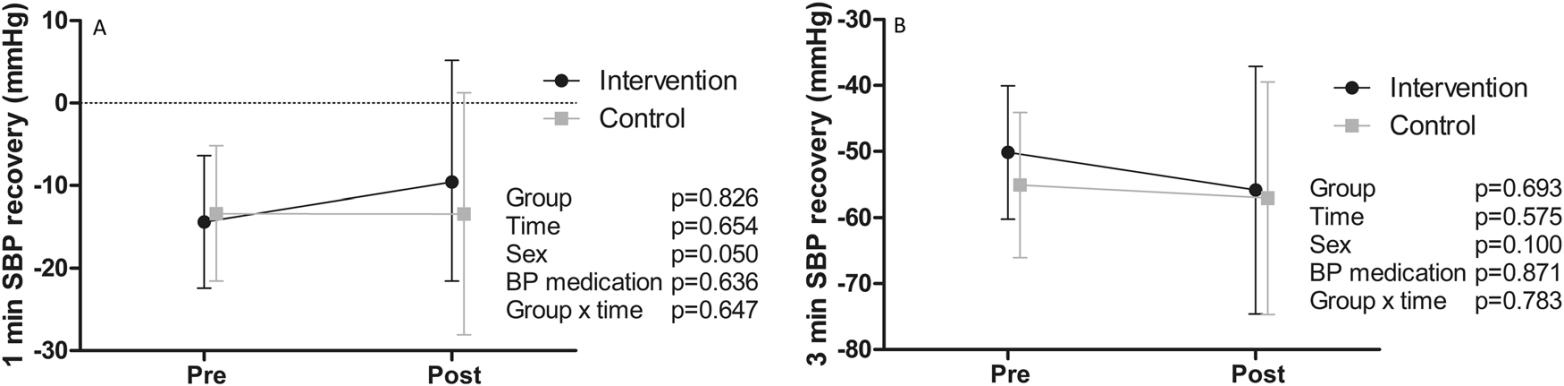
Intervention effects on blood pressure recovery. Systolic blood pressure recovery one minute (**A**) and three minutes (**B**) after maximal exercise test in the intervention (black dots) and control (grey squares) groups before (pre) and after (post) the intervention. Estimates are model-based least squares means and error bars represent 95% confidence intervals. SBP systolic blood pressure, BP blood pressure.

None of the changes in BP variables from baseline to six months were statistically significantly different between groups (Fig. 1). Resting SBP and maximal DBP were higher in the INT group compared to the CON group throughout the intervention. Resting SBP and DBP decreased in both groups during the intervention. SBP, but not DBP, at submaximal exercise intensities was higher in the INT, but no significant changes during the intervention were observed in SBP or DBP during submaximal exercise (Fig. 2). SBP recovery at one and three minutes after exercise termination was similar in both groups with no changes during the intervention (Fig. 3).

### Additional analyses

When the main analyses were replicated using group division according to the change in SB, all of the results remained practically identical to the results with the original group allocation (Supplementary Tables 1–3). However, when dividing the group based on the change in total PA, DBP during submaximal intensity exercise tended to decrease in the more active group, whereas the change was smaller or even opposite in the less active group, albeit statistically significant changes at a given intensity were present for neither group (group x intensity x time *p* = 0.025; all pairwise comparisons *p* > 0.05). Furthermore, SBP during the exercise test trended towards a decrease in the more active group. However, the changes in SBP during the exercise test were not statistically significant (group x intensity x time *p* = 0.075). All of the estimates and *p*-values for the total PA based groups are shown in Supplementary Tables 4–6.

### Correlations of changes during the intervention

In the whole study group, the change in standing percentage of accelerometer wear time was positively associated with the change in SBP/MET-slope (*r* = 0.29, *p* = 0.043), and the association remained statistically significant when adjusting for the change in BMI (*r* = 0.36, *p* = 0.014). No other significant associations between changes in accelerometer outcomes and resting, maximal, or recovery BP were found (data not shown).

**Fig. 4:**
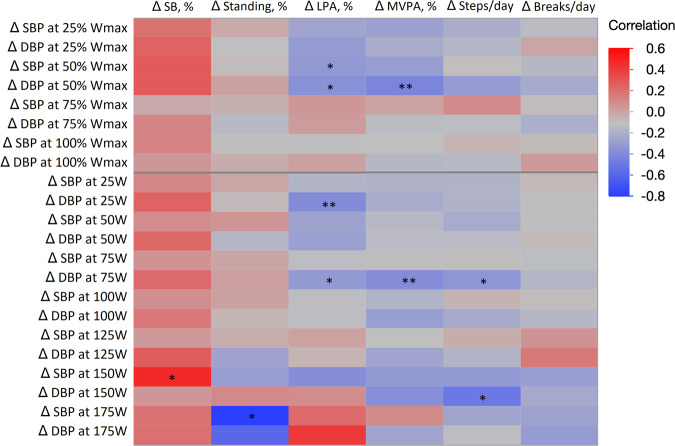
Heat map of Pearson’s correlations of changes (Δ) in the whole study group in the accelerometry and blood pressure variables during exercise test at relative intensities (i.e., percentage of maximal power output) and at absolute workloads (W). Accelerometry variables were analysed as proportions of daily accelerometer wear time. SBP = systolic blood pressure, DBP = diastolic blood pressure, % Wmax percentage of maximal power output, SB sedentary behaviour, LPA light physical activity, MVPA moderate-to-vigorous physical activity. *p < 0.05, **p < 0.01.

When investigating the associations between the changes in the accelerometer variables and BP at submaximal relative (% of Wmax) and absolute (W) exercise intensities, the changes in LPA and MVPA percentages of accelerometer wear time were associated with the changes in BP at relative intensities of 25 and 50% of Wmax. Corresponding findings were present for absolute exercise intensities. Furthermore, the changes in daily steps were correlated with 2 out of 22 possible variables and standing and SB with 1 out of 22. A heat map of the non-adjusted correlations is presented in Fig. 4 (the corresponding correlation coefficients and *p*-values are presented in Supplementary Table 7). When adjusting for the change in BMI, correlations remained similar to the non-adjusted correlations (presented in Supplementary Table 8).

Additionally, the change in BMI correlated with the change in SBP at 25% of Wmax (*r* = 0.36, *p* = 0.014), and at 50 W, 100 W, and 125 W (*r* = 0.29, 0.30, and 0.33, *p* = 0.036, 0.030, and 0.035, respectively). Furthermore, the change in BMI correlated with the changes in SBP/MET-slope (*r* = 0.35, *p* = 0.009) and 1 min SBP (*r* = −0.49, *p* = 0.046). The change in body fat percentage correlated with the change in SBP at 150 W (*r* = 0.46, *p* = 0.028).

## Discussion

In the present study, we found that a six-month intervention that was aimed to reduce daily SB by 1 h did not affect BP at rest or during or after incremental maximal exercise test among sedentary adults with metabolic syndrome. However, the changes in device-measured LPA or MVPA were inversely correlated with changes in BP at submaximal intensities, suggesting that increasing daily LPA or MVPA may lower BP (mainly DBP) at submaximal (~light-to-moderate) exercise intensities.

To date, to the best of our knowledge, this is the first study to comprehensively investigate the effects of modifying daily SB and PA on BP at rest as well as during submaximal, maximal and recovery from physical exercise in an interventional setting. Previous studies have reported reductions in resting BP (mean arterial pressure reduction from 3 to 10 mmHg, mostly driven by DBP reduction) with a workplace-based intervention that focused on breaking up prolonged SB using a computer application encouraging non-exercise PA [11, 12]. However, these studies implemented a very different intervention and measurements compared to the present study (e.g., not controlling for PA or not directly aiming at reducing SB).

The present results are similar to those we found at the midpoint of the same intervention, showing that resting BP was unchanged between groups after three months [25]. Similarly, a non-randomised three-month intervention that focused on reducing SB with or without increasing exercise training did not find any differences in resting BP compared to a control group [26]. However, similar to our study, even though no main effects of time x intervention were present, a tendency towards lower BP after the intervention was present for all groups [26]. In our study, the decrease in both groups’ BP might be explained, in part, by the increased steps in both groups.

In our study, maximal BP during exercise and BP during recovery from maximal exercise did not change differently between groups during the intervention. We believe that the low-intensity intervention—focused only on reducing SB by replacing it with standing and non-exercise PA—is not sufficient to alter the physiological responses to maximal exercise, despite a significant increase in MVPA in the INT group. Remarkably, the MVPA in this study consisted practically entirely of moderate-intensity PA, the median vigorous PA being only 0.6 min/day during the intervention [19]. However, maximal BP is closely related to cardiorespiratory fitness – individuals with higher fitness also tend to have higher maximal BP [27]. In agreement with that, we have previously shown in the same study group that the intervention did not improve cardiorespiratory fitness [21].

The additional analyses in this study using group divisions based on the measured changes in SB strengthen the conclusion that a mere SB-reduction intervention is not sufficient at influencing BP at rest, during submaximal or maximal exercise or recovery from maximal exercise. Interestingly, it seems that increasing LPA, MVPA or a combination of them might decrease BP during light-to-moderate intensity PA. This is supported by the observation that the changes in LPA and MVPA correlated inversely with BP during PA of such intensity (Fig. 4). However, after adjusting the correlation analyses for changes in BMI, the changes in PA outcomes (with the exception of standing) were associated only with changes in DBP at different submaximal intensities (Supplementary Table 8).

The reduction in submaximal BP when LPA or MVPA are increased could be related to blunting of the (exaggerated) exercise pressor reflex, i.e., decreased sympathetic activity. Why this phenomenon would be more pronounced for diastolic blood pressure (which remained significant after BMI adjustment), could, speculatively, be related to the adverse effects of adiposity on arterial elastance, which is closely related to systolic blood pressure. Thus, the possible beneficial effects of increased PA might show a stronger association with DBP rather than SBP during submaximal exercise in a sample of adults with overweight or obesity. Further, as DBP often decreases with increasing exercise intensity reflecting decreased peripheral vascular resistance, reduced DBP during exercise may indicate that vasodilatory reactions may also have improved or vasoconstrictive reactions reduced during submaximal exercise.

Furthermore, when dividing the participants according to the change in total PA (i.e., LPA + MVPA) submaximal exercise BP tended to decrease in the more active group while the less active group showed no beneficial changes in BP. However, even though the whole model for DBP during the exercise test was significant (group × intensity × time *p* = 0.025), the pairwise comparisons failed to reach statistical significance. This is likely due to insufficient statistical power to detect changes in the post hoc comparisons.

A previous randomised crossover trial demonstrated that supervised light-intensity exercise training (i.e., 33% of heart rate reserve) three times per week for 10 weeks, 50 min per session, was successful at reducing submaximal exercise SBP [28]. Our results add to this finding by suggesting that unsupervised non-exercise PA may be associated with similar benefits. Unfortunately, the previous study did not report DBP values during exercise [28]. Moreover, the participants were leaner compared to the participants in our study, which, in the light of our BMI-adjusted analyses, might explain why they found significant effects on SBP and we did not [28]. The adverse effects of excess adiposity on SBP may overcome the benefits of light or moderate exercise. Indeed, we observed that decreases in BMI during the intervention were associated with decreases in SBP during submaximal exercise. Furthermore, a negative correlation between the change in BMI and the change in 1 min SBP recovery was observed, indicating a faster SBP recovery when BMI decreased.

BP during light-to-moderate intensity PA is important as adults spend almost 4.5 h/day, on average, in LPA or MVPA [3]. Higher BP at intensities corresponding to daily activities (i.e., light-intensity walking, cleaning, or cooking) seems to have similar effects on the heart and blood vessels as resting hypertension, such as increased left ventricular mass index and arterial stiffness, regardless of resting BP [29, 30]. Therefore, increasing daily LPA or MVPA could potentially improve cardiovascular health by lowering BP during submaximal PA. However, further studies are needed to confirm this result.

### Strengths and weaknesses

Strengths of this study include the accelerometer measurement during the whole intervention with six-second intervals and using validated algorithms for accelerometer data analysis. Moreover, the intervention was successful at reducing SB in the INT group as intended. Previous SB reduction interventions have also achieved similar, −40 min/day changes in SB [31]. However, as sleep was not assessed, full 24 h movement behaviours cannot be estimated although the analyses were adjusted for accelerometer wear time [19]. Additionally, sleep time may itself affect BP and thus, future studies should include sleep assessments.

Moreover, the present analysis was based on secondary outcomes, and therefore the study was likely underpowered to detect changes in BP. The sample size (*n* = 64) was calculated according to the primary outcome of the trial (whole-body insulin sensitivity) [19]. A study protocol for a comparable SB reduction intervention that aims at improving BP calculated that a sample size of 300 participants (150 per group) would achieve 80% statistical power to detect a statistically significant (alfa set at 5%) between-group difference of 4 mmHg in SBP [32]. This calculation assumed a 20% drop-out rate (i.e., 240 completers) and SDs of 10 and 11 mmHg for baseline and change SBP [32].

Excluding participants with SBP ≥160 and/or DBP ≥100 mmHg may have affected the results as hypotensive effects are generally more pronounced in participants with higher baseline BP. The comprehensive BP assessment using both resting, exercise until exhaustion and recovery values from maximal exercise provides a broad picture of the functioning of the cardiovascular system. Yet, no out-of-office measurements (such as 24-h ambulatory BP measurement or BP home monitoring) were performed. The potential benefits of home-based measures in comparison to clinic-based measurement are the unmasking of masked hypertension and elimination of the white-coat effect [33], the first of which is also achieved using BP measurement during exercise testing [13]. However, individuals with white-coat hypertension still seem to have higher BP during exercise than individuals with normotension [34], but this might simply be descriptive of the increased cardiovascular risks associated with white-coat hypertension [35].

### Future perspectives

Based on the secondary analyses of the present randomised controlled trial, investigating the effects of a LPA- or moderate PA-based intervention on BP during daily free-living light-to-moderate intensity activities (i.e., using a 24-h ambulatory BP measurement with simultaneous accelerometry) would be justified. Our results suggest that non-exercise LPA or MVPA could lower 24-h ambulatory BP, which is affected by PA during the day.

## Conclusion

A six-month intervention aimed at reducing SB by 1 h/day does not have an effect on BP at rest, during submaximal or maximal exercise, or during recovery from maximal exercise. However, based on the secondary analyses of the data, increasing daily LPA or MVPA might improve BP, especially DBP, during light-to-moderate intensity PA.

## Summary

### What is known about the topic

- High sedentary behaviour is associated with higher blood pressure in previous studies.
- In addition to resting blood pressure, blood pressure during and after physical exercise is associated with future cardiovascular risk.

### What this study adds

- A free-living intervention aimed at reducing sedentary behaviour by one hour per day for six months did not influence blood pressure at rest or during or after incremental maximal exercise test in sedentary adults with metabolic syndrome. However, the sample size in this study might have been a limitation and thus the study is regarded as an exploratory randomised controlled trial (RCT).
- Succeeding at increasing light-to-moderate physical activity may nevertheless lower blood pressure during physical activity of corresponding intensity (i.e., household tasks) according to the results of the present study.

### Supplementary information

Supplemental material

## Data Availability

The data for this study is available for a reasonable request from the corresponding author.
