# Supplementary material for: Effects of reduced sedentary time on resting, exercise and post-exercise blood pressure in inactive adults with metabolic syndrome – a six-month exploratory RCT
Source: J Hum Hypertens. 2024 Jan 24;38(4):314–21. doi: 10.1038/s41371-024-00894-6 (PMC11001575; doi:10.1038/s41371-024-00894-6)
Supplement: Supplementary file 1 — Supplemental material [file 41371_2024_894_MOESM1_ESM.docx]

**Effects of reduced sedentary time on resting, exercise and post-exercise blood pressure in inactive adults with metabolic syndrome – a six-month exploratory RCT**

**Supplementary file**

Jooa Norha^1*^, Tanja Sjöros^1^, Taru Garthwaite^1^, Saara Laine^1^, Maria Saarenhovi^2^, Petri Kallio^2, 3^, Kirsi Laitinen^4^, Noora Houttu^4^, Henri Vähä-Ypyä^5^, Harri Sievänen^5^, Eliisa Löyttyniemi^6^, Tommi Vasankari^5, 7^, Juhani Knuuti^1^, Kari K. Kalliokoski^1^, Ilkka H. A. Heinonen^1, 8^

1 Turku PET Centre, University of Turku and Turku University Hospital, Turku, Finland

2 Department of Clinical Physiology and Nuclear Medicine, University of Turku and Turku University Hospital, Turku, Finland

3 Paavo Nurmi Centre and Unit for Health and Physical Activity, University of Turku, Turku, Finland

4 Institute of Biomedicine, University of Turku, Turku, Finland

5 The UKK Institute for Health Promotion Research, Tampere, Finland

6 Department of Biostatistics, University of Turku and Turku University Hospital, Turku, Finland

7 Faculty of Medicine and Health Technology, Tampere University, Tampere, Finland

8 Rydberg Laboratory of Applied Sciences, University of Halmstad, Halmstad, Sweden

*Corresponding author: Jooa Norha, Turku PET Centre, University of Turku and Turku University Hospital, P.O. Box 52, 20521 Turku, Finland, tel. +358 443312942, E-mail: [jooa.norha@utu.fi](mailto:jooa.norha@utu.fi)


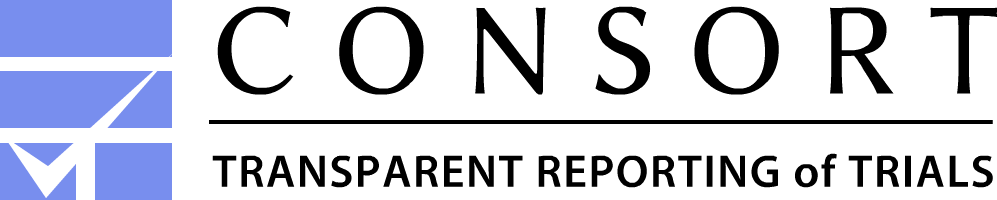


Supplementary Figure 1.

**CONSORT 2010 Flow Diagram**

Allocated to intervention group (n=33)

♦ Received allocated intervention (n=33)

Allocated to control group (n=31)

♦ Received allocated intervention (n=31)

Analysed (n=31)
♦ Valid accelerometer data (n=28)

♦ Complete maximal exercise test data (n=29)

♦ Complete resting blood pressure data (n=28)

Analysed (n=33)
♦ Valid accelerometer data (n=29)

♦ Complete maximal exercise test data (n=28)

♦ Complete resting blood pressure data (n=31)

Participated in follow-up measurements (n=32)

♦ Discontinued for personal reasons (n=1)

Participated in follow-up measurements (n=28)

Discontinued intervention (n=3)

♦ Personal reasons (n=1)

♦ Low back pain (n=1)

## Follow-Up

## Analysis

## Enrollment

## Allocation

Randomized (n=64)

Assessed for eligibility (n=263)

Excluded at initial contact (n=112)

♦  Not meeting inclusion criteria based on interview (n=112)

Excluded after screening (n=87)

♦ No metabolic syndrome (n=45)

♦ Not meeting accelerometry criteria (n=13)

♦ Comorbidities (n=10)

♦  Declined to participate (n=9)

♦ Previous exposure to radiation (n=2)

♦  Died during screening (n=1)

Supplementary Table 1. Resting, maximal exercise and recovery blood pressures before and after the intervention in the sedentary behavior (SB) reduction group and the continuously sedentary group.

|  | Less sedentary |  | Continuously sedentary |  | Difference | Group x time p-value | p-values |
| --- | --- | --- | --- | --- | --- | --- | --- |
|  | Pre | Post | Pre | Post |  |  |  |
| Resting SBP, mmHg | 143.55 (138.07, 149.02) | 140.48 (134.84, 146.12) | 141.38 (135.62, 147.15) | 136.54 (130.51, 142.57) | 1.78 (-4.59, 8.14) | 0.578 | Group 0.416, BPmed 0.786, **Time 0.016**, Sex 0.423 |
| Resting DBP, mmHg | 88.09 (85.15, 91.03) | 87.17 (84.12, 90.22) | 88.62 (85.52, 91.71) | 85.29 (82.01, 88.57) | 2.41 (-1.58, 6.40) | 0.231 | Group 0.730, BPmed 0.976, **Time 0.037**, Sex 0.936 |
| Maximal SBP, mmHg | 221.75 (213.64, 229.86) | 218.29 (210.20, 226.39) | 211.74 (203.21, 220.27) | 211.34 (202.64, 220.22) | -3.15 (-12.41, 6.11) | 0.498 | Group 0.126, BPmed 0.923, Time 0.418, Sex 0.217 |
| Maximal DBP, mmHg | 92.98 (88.91, 97. 04) | 92.70 (88.65, 96.75) | 87.90 (83.72, 92.09) | 88.92 (84.44, 93.41) | -1.29 (-8.15, 5.67) | 0.707 | Group 0.074, **BPmed 0.033**, Time 0.829**, Sex 0.021** |
| Change from rest to maximal SBP, mmHg | 82.85 (75.10, 90.59) | 77.40 (69.68, 85.13) | 77.25 (69.11, 85.38) | 74.32 (65.90, 82.73) | -2.51 (-12.06, 7.04) | 0.600 | Group 0.400, BPmed 0.364, Time 0.084**, Sex 0.019** |
| Change from rest to maximal DBP, mmHg | 4.98 (1.06, 8.90) | 3.19 (-0.71, 7.09) | 1.49 (-2.54, 5.52) | 1.83 (-2.50, 6.17) | -2.13 (-9.15, 4.89) | 0.546 | Group 0.291**, BPmed 0.019**, Time 0.681, Sex 0.069 |
| SBP/MET-slope, mmHg/MET | 15.30 (13.56, 17.05) | 14.78 (13.05, 16.52) | 14.35 (12.50, 16.20) | 13.42 (11.51, 15.33) | 0.41 (-2.34, 3.17) | 0.765 | Group 0.289, BPmed 0.537, Time 0.294, Sex 0.388 |
| SBP change at 1 min recovery, mmHg | -13.93 (-21.99, -5.86) | -8.42 (-22.90, 6.05) | -13.84 (-21.87, -5.80) | -15.21 (-30.36, -0.06) | 6.88 (-14.78, 28.54) | 0.518 | Group 0.591, BPmed 0.640, Time 0.697, **Sex 0.048** |
| SBP change at 3 min recovery, mmHg | -52.85 (-63.51, -42.20) | -53.91 (-71.91, -35.92) | -52.05 (-62.67, -41.42) | -59.28 (-77.33, -41.23) | 6.17 (-20.90, 33.25) | 0.646 | Group 0.769, BPmed 0.911, Time 0.543, Sex 0.091 |

Reported as model-based mean (95% CI), adjusted for sex and blood pressure medication. Statistically significant results are bolded. SBP = systolic blood pressure, DBP = diastolic blood pressure, MET = metabolic equivalent of task, BPmed = blood pressure medication, SB = sedentary behavior. Less sedentary = SB reduction of ≥3 percentage points of accelerometer wear time. Continuously sedentary = SB reduction <3 percentage points or an increase in SB.

Supplementary Table 2. Intervention effects on systolic blood pressure at rest before the exercise test and during exercise at 25%, 50%, 75% and 100% of maximal power output (Wmax) when dividing the study group according to the change in sedentary behavior (SB).

|  | Less sedentary |  | Continuously sedentary |  |
| --- | --- | --- | --- | --- |
|  | Pre | Post | Pre | Post |
| Rest, mmHg | 141.43 (134.85, 148.00) | 142.99 (135.94, 150.03) | 136.52 (129.58, 143.46) | 138.63 (130.87, 146.38) |
| 25% Wmax, mmHg | 168.37 (161.55, 175.19) | 165.70 (158.59, 172.81) | 158.21 (150.87, 165.54) | 157.50 (149.49, 165.51) |
| 50% Wmax, mmHg | 187.60 (180.87, 194.34) | 182.30 (175.23, 189.37) | 175.81 (168.49, 183.12) | 172.23 (164.35, 180.11) |
| 75% Wmax, mmHg | 205.42 (198.60, 212.24) | 204.80 (197.65, 211.94) | 192.65 (185.35, 199.95) | 187.87 (179.95, 195.79) |
| 100% Wmax, mmHg | 221.59 (214.88, 228.31) | 217.69 (210.58, 224.80) | 212.03 (204.95, 219.10) | 211.55 (203.74, 219.35) |

Reported as model-based mean (95% CI), adjusted for sex and blood pressure medication. SB = Sedentary behavior. Less sedentary = SB reduction of ≥3 percentage points of accelerometer wear time. Continuously sedentary = SB reduction <3 percentage points or an increase in SB.

Group x time x intensity p = 0.494
Group x time p = 0.851
Group x intensity p = 0.104
Time x intensity p = 0.053
Group p = 0.018
Time p = 0.327
Sex p = 0.692
Intensity p < 0.001
Blood pressure medication p = 0.274

Supplementary Table 3. Intervention effects on diastolic blood pressure at rest before the exercise test and during exercise at 25%, 50%, 75% and 100% of maximal power output (Wmax) when dividing the study group according to the change in sedentary behavior (SB).

|  | Less sedentary |  | Continuously sedentary |  |
| --- | --- | --- | --- | --- |
|  | Pre | Post | Pre | Post |
| Rest, mmHg | 88.06 (84.51, 91.62) | 90.27 (86.46, 94.08) | 86.88 (83.13, 90.64) | 86.94 (82.68, 91.21) |
| 25% Wmax, mmHg | 91.55 (87.82, 95.28) | 90.93 (87.09, 94.77) | 87.59 (83.58, 91.61) | 90.26 (85.86, 94.66) |
| 50% Wmax, mmHg | 92.80 (89.13, 96.48) | 91.91 (88.07, 95.75) | 87.11 (83.09, 91.12) | 89.92 (85.56, 94.28) |
| 75% Wmax, mmHg | 92.48 (88.75, 96.21) | 91.44 (87.58, 95.31) | 88.81 (84.80, 92.82) | 87.75 (83.39, 92.10) |
| 100% Wmax, mmHg | 93.68 (90.01, 97.35) | 93.10 (89.24, 96.97) | 88.42 (84.59, 92.25) | 88.80 (84.49, 93.11) |

Reported as model-based mean (95% CI), adjusted for sex and blood pressure medication. SB = sedentary behavior. Less sedentary = SB reduction of ≥3 percentage points of accelerometer wear time. Continuously sedentary = SB reduction <3 percentage points or an increase in SB.

Group x time x intensity p = 0.309
Group x time p = 0.655
Group x intensity p = 0.628
Time x intensity p = 0.585
Group p = 0.091
Time p = 0.760
Sex p = 0.101
Intensity p = 0.022
Blood pressure medication p = 0.206

Supplementary Table 4. Resting, maximal exercise and recovery blood pressures before and after the intervention in the more active (according to change in total physical activity) group and the less active group.

|  | More active |  | Less active |  | Difference | Group x time p-value | p-values |
| --- | --- | --- | --- | --- | --- | --- | --- |
|  | Pre | Post | Pre | Post |  |  |  |
| Resting SBP, mmHg | 142.17 (137.33, 147.01) | 138.87 (133.61, 144.14) | 142.92 (137.94, 147.90) | 138.44 (133.35, 143.53) | 1.18 (-7.52, 9.88) | 0.787 | Group 0.941**, Time 0.018**, Sex 0.453, BPmed 0.749 |
| Resting DBP, mmHg | 87.46 (84.76, 90.16) | 86.90 (83.88, 89.92) | 89.36 (86.56, 92.16) | 85.80 (82.90) | 3.00 (-2.37, 8.37) | 0.269 | Group 0.757**, Time 0.045**, Sex 0.864, BPmed 0.983 |
| Maximal SBP, mmHg | 218.54 (211.26, 225.82) | 214.32 (206.58, 222.07) | 215.29 (207.88, 222.69) | 215.76 (208.40, 223.12) | -4.69 (-17.54, 8.16) | 0.468 | Group 0.766, Time 0.422, Sex 0.323, BPmed 0.914 |
| Maximal DBP, mmHg | 90.13 (86.06, 94.21) | 90.09 (85.51, 94.67) | 90.76 (86.61, 94.91) | 91.79 (87.36, 96.22) | -1.07 (-9.98, 7.83) | 0.811 | Group 0.585, Time 0.774**, Sex 0.022, BPmed 0.036** |
| Change from rest to maximal SBP, mmHg | 82.59 (75.65, 89.54) | 72.25 (64.80, 79.70) | 77.05 (69.96, 84.13) | 79.25 (72.19, 86.31) | 12.54 (-25.29, 0.20) | 0.054 | Group 0.810, Time 0.084, Sex 0.094, BPmed 0.370 |
| Change from rest to maximal DBP, mmHg | 3.82 (-0.09, 7.73) | 0.26 (-4.23, 4.75) | 2.30 (-1.69, 6.29) | 4.71 (0.37, 9.05) | -5.97 (-14.68, 2.92) | 0.185 | Group 0.489, Time 0.743, **Sex 0.020, BPmed 0.016** |
| SBP/MET-slope, mmHg/MET* | 15.06 (13.41, 16.91) | 12.51 (11.00, 14.22) | 13.46 (11.93, 15.38) | 13.95 (12.37, 15.74) | 3.04 (2.85, 3.05) | 0.068 | Group 0.979, Time 0.096, Sex 0.318, BPmed 0.725 |
| SBP change at 1 min recovery, mmHg | -10.91 (-19.08, -2.75) | 1.32 (-11.35, 13.99) | -15.57 (-24.63, -6.51) | -21.48 (-31.95, -11.00) | 19.14 (-3.25, 39.53) | 0.095 | **Group 0.008**, Time 0.470, Sex 0.198, BPmed 0.590 |
| SBP change at 3 min recovery, mmHg | -47.52 (-59.66, -35.38) | -46.72 (-63.23, -30.22) | -57.96 (-72.39, -43.53) | -65.33 (-79.14, -51.52) | 8.16 (-20.51, 36.83) | 0.569 | Group 0.058, Time 0.610, Sex 0.210, BPmed 0.790 |

Reported as model-based mean (95% CI), adjusted for sex and blood pressure medication. Statistically significant results are bolded. *Log10 transformation was used to ensure normal distribution of the residuals; the presented estimates are back-transformed geometric means. More
active = total physical activity (PA) increase of ≥3 percentage points of accelerometer wear time. Less active = total PA increase <3 percentage points or a decrease in total PA. SBP = systolic blood pressure, DBP = diastolic blood pressure, MET = metabolic equivalent of task, BPmed = blood pressure medication.

Supplementary Table 5. Intervention effects on systolic blood pressure at rest before the exercise test and during exercise at 25%, 50%, 75% and 100% of maximal power output (Wmax) when dividing the study group according to the change in total physical activity.

|  | More active |  | Less active |  |
| --- | --- | --- | --- | --- |
|  | Pre | Post | Pre | Post |
| Rest, mmHg | 136.70 (129.94, 143.47) | 139.82 (132.41, 147.24) | 141.81 (134.71, 148.91) | 142.25 (134.34, 150.16) |
| 25% Wmax, mmHg | 165.85 (158.70, 173.00) | 160.22 (152.59, 167.86) | 161.70 (154.38, 169.02) | 164.07 (156.09, 172.04) |
| 50% Wmax, mmHg | 183.35 (176.35, 190.35) | 175.41 (167.89, 182.93) | 180.95 (173.59, 188.31) | 180.46 (172.53, 188.40) |
| 75% Wmax, mmHg | 199.26 (192.22, 206.30) | 195.86 (188.30, 203.42) | 199.78 (192.37, 207.19) | 198.71 (190.69, 206.73) |
| 100% Wmax, mmHg | 217.09 (210.16, 224.02) | 212.71 (205.20 (220.22) | 217.31 (210.11, 224.52) | 217.36 (209.43, 225.30) |

Reported as model-based mean (95% CI), adjusted for sex and blood pressure medication. More active = total physical activity (PA) increase of ≥3 percentage points of accelerometer wear time. Less active = total PA increase <3 percentage points or a decrease in total PA.

Group x time x intensity p = 0.079
Group x time p = 0.289
Group x intensity p = 0.892
Time x intensity p = 0.072
Group p = 0.663
Time p = 0.355
Sex p = 0.622
Intensity p < 0.001
Blood pressure medication p = 0.362

Supplementary Table 6. Intervention effects on diastolic blood pressure at rest before the exercise test and during exercise at 25%, 50%, 75% and 100% of maximal power output (Wmax) when dividing the study group according to the change in total physical activity.

|  | More active |  | p-value | Less active |  | p-value |
| --- | --- | --- | --- | --- | --- | --- |
|  | Pre | Post |  | Pre | Post |  |
| Rest, mmHg | 86.81 (83.15, 90.48) | 89.05 (85.15, 92.95) | 0.364 | 88.23 (84.39, 92.07) | 88.47 (84.29, 92.64) | 0.923 |
| 25% Wmax, mmHg | 89.76 (85.84, 93.68) | 88.56 (84.55, 92.56) | 0.647 | 89.59 (85.58, 93.59) | 92.54 (88.33, 96.75) | 0.245 |
| 50% Wmax, mmHg | 91.01 (87.18, 94.83) | 89.59 (85.62, 93.57) | 0.590 | 89.27 (85.23, 93.31) | 92.42 (88.21, 96.63) | 0.227 |
| 75% Wmax, mmHg | 90.96 (87.11, 94.82) | 90.67 (86.70, 94.65) | 0.911 | 90.51 (86.43, 94.58) | 88.72 (84.48, 92.96) | 0.563 |
| 100% Wmax, mmHg | 91.40 (87.61, 95.19) | 92.82 (88.85, 96.78) | 0.720 | 90.94 (87.04, 94.85) | 89.40 (85.19, 93.61) | 0.576 |

Reported as model-based mean (95% CI), adjusted for sex and blood pressure medication. Pairwise comparisons are uncorrected. More active = total physical activity (PA) increase of ≥3 percentage points of accelerometer wear time. Less active = total PA increase <3 percentage points or a decrease in total PA.

Group x time x intensity p = 0.025
Group x time p = 0.854
Group x intensity p = 0.445
Time x intensity p = 0.603
Group p = 0.957
Time p = 0.801
Sex p = 0.165
Intensity p = 0.107
Blood pressure medication p = 0.292

Supplementary Table 7. Pearson’s correlations of changes (Δ) in the whole study group in the accelerometry and blood pressure variables during exercise test at relative intensities (i.e., percentage of maximal power output) and at absolute workloads (W).

|  |  | Δ SB, % | | Δ Standing, % | Δ LPA, % | | | Δ MVPA, % | | Δ Steps/day | | Δ Breaks/day |
| --- | --- | --- | --- | --- | --- | --- | --- | --- | --- | --- | --- | --- |
| Δ SBP at 25% Wmax | r | 0.204 | | -0.012 | -0.245 | | | -0.272 | | -0.204 | | -0.128 |
|  | p-value | 0.206 | | 0.941 | 0.128 | | | 0.089 | | 0.206 | | 0.430 |
| Δ DBP at 25% Wmax | r | 0.259 | | -0.105 | -0.299 | | | -0.214 | | -0.154 | | 0.000 |
|  | p-value | 0.106 | | 0.517 | 0.061 | | | 0.186 | | 0.344 | | 0.999 |
| Δ SBP at 50% Wmax | r | 0.290 | | -0.080 | **-.315*** | | | -0.292 | | -0.092 | | -0.147 |
|  | p-value | 0.059 | | 0.609 | **0.040** | | | 0.057 | | 0.557 | | 0.346 |
| Δ DBP at 50% Wmax | r | 0.295 | | 0.019 | **-.351*** | | | **-.420**** | | -0.296 | | -0.222 |
|  | p-value | 0.055 | | 0.902 | **0.021** | | | **0.005** | | 0.054 | | 0.153 |
| Δ SBP at 75% Wmax | r | -0.010 | | -0.035 | 0.064 | | | 0.015 | | 0.109 | | -0.081 |
|  | p-value | 0.951 | | 0.835 | 0.700 | | | 0.928 | | 0.509 | | 0.625 |
| Δ DBP at 75% Wmax | r | 0.127 | | -0.140 | 0.038 | | | -0.122 | | -0.117 | | -0.192 |
|  | p-value | 0.442 | | 0.394 | 0.816 | | | 0.461 | | 0.477 | | 0.241 |
| Δ SBP at 100% Wmax | r | 0.139 | | -0.109 | -0.108 | | | -0.098 | | -0.052 | | -0.078 |
|  | p-value | 0.341 | | 0.458 | 0.459 | | | 0.504 | | 0.721 | | 0.594 |
| Δ DBP at 100% Wmax | r | 0.057 | | -0.023 | 0.020 | | | -0.148 | | -0.139 | | 0.050 |
|  | p-value | 0.702 | | 0.879 | 0.893 | | | 0.322 | | 0.351 | | 0.738 |
| Δ SBP at 25W | r | 0.123 | | 0.004 | -0.169 | | | -0.178 | | -0.175 | | -0.068 |
|  | p-value | 0.414 | | 0.981 | 0.260 | | | 0.237 | | 0.244 | | 0.655 |
| Δ DBP at 25W | r | 0.262 | | -0.078 | **-.383**** | | | -0.210 | | -0.174 | | -0.109 |
|  | p-value | 0.079 | | 0.606 | **0.009** | | | 0.161 | | 0.248 | | 0.473 |
| Δ SBP at 50W | r | 0.103 | | 0.071 | -0.258 | | | -0.144 | | -0.214 | | -0.110 |
|  | p-value | 0.486 | | 0.632 | 0.077 | | | 0.329 | | 0.144 | | 0.459 |
| Δ DBP at 50W | r | 0.242 | | -0.153 | -0.274 | | | -0.134 | | -0.134 | | -0.072 |
|  | p-value | 0.097 | | 0.299 | 0.060 | | | 0.365 | | 0.362 | | 0.626 |
| Δ SBP at 75W | r | 0.075 | | 0.009 | -0.114 | | | -0.109 | | -0.087 | | -0.118 |
|  | p-value | 0.611 | | 0.951 | 0.439 | | | 0.460 | | 0.556 | | 0.426 |
| Δ DBP at 75W | r | 0.231 | | 0.027 | **-.310*** | | | **-.377**** | | **-.327*** | | -0.152 |
|  | p-value | 0.114 | | 0.856 | **0.032** | | | **0.008** | | **0.023** | | 0.302 |
| Δ SBP at 100W | r | 0.095 | | 0.018 | -0.114 | | | -0.172 | | -0.051 | | -0.083 |
|  | p-value | 0.537 | | 0.904 | 0.456 | | | 0.260 | | 0.738 | | 0.588 |
| Δ DBP at 100W | r | 0.180 | | -0.053 | -0.116 | | | -0.281 | | -0.220 | | -0.150 |
|  | p-value | 0.243 | | 0.735 | 0.455 | | | 0.064 | | 0.152 | | 0.330 |
| Δ SBP at 125W | r | 0.052 | | -0.032 | 0.014 | | | -0.094 | | -0.023 | | 0.072 |
|  | p-value | 0.762 | | 0.853 | 0.933 | | | 0.584 | | 0.893 | | 0.675 |
| Δ DBP at 125W | r | 0.282 | | -0.255 | -0.055 | | | -0.244 | | -0.170 | | 0.179 |
|  | p-value | 0.100 | | 0.139 | 0.753 | | | 0.158 | | 0.328 | | 0.303 |
| Δ SBP at 150W | r | **.489*** | | -0.287 | -0.372 | | | -0.313 | | -0.311 | | -0.282 |
|  | p-value | **0.029** | | 0.221 | 0.106 | | | 0.179 | | 0.182 | | 0.228 |
| Δ DBP at 150W | r | 0.063 | | 0.114 | 0.097 | | | -0.365 | | **-.492*** | | -0.222 |
|  | p-value | 0.791 | | 0.633 | 0.685 | | | 0.114 | | **0.028** | | 0.346 |
| Δ SBP at 175W | r | 0.193 | | **-.808*** | 0.231 | | | 0.105 | | -0.247 | | -0.260 |
|  | p-value | 0.679 | | **0.028** | 0.618 | | | 0.822 | | 0.593 | | 0.574 |
| Δ DBP at 175W | r | 0.205 | | -0.582 | 0.416 | | | -0.244 | | -0.116 | | -0.296 |
|  | p-value | 0.660 | | 0.171 | 0.353 | | | 0.598 | | 0.805 | | 0.519 |
|  |  |  |  | | |  |  | |  | |  | |

Accelerometry variables were analysed as proportions of daily accelerometer wear time. SBP = systolic blood pressure, DBP = diastolic blood pressure, % Wmax = percentage of maximal power output, SB = sedentary behavior, LPA = light physical activity, MVPA = moderate-to-vigorous physical activity. * p<0.05. Statistically significant correlations are bolded.

Supplementary Table 8. Pearson’s correlations of changes (Δ) in the whole study group in the accelerometry and blood pressure variables during exercise test at relative intensities (i.e., percentage of maximal power output) and at absolute workloads (W), adjusted for Δ body mass index.

|  |  | Δ SB, % | | Δ Standing, % | Δ LPA, % | | | Δ MVPA, % | | Δ Steps/day | | Δ Breaks/day |
| --- | --- | --- | --- | --- | --- | --- | --- | --- | --- | --- | --- | --- |
| Δ SBP at 25% Wmax | r | 0.121 | | 0.028 | -0.128 | | | -0.223 | | -0.134 | | -0.066 |
|  | p-value | 0.463 | | 0.865 | 0.436 | | | 0.172 | | 0.417 | | 0.689 |
| Δ DBP at 25% Wmax | r | 0.234 | | -0.092 | -0.271 | | | -0.194 | | -0.127 | | 0.026 |
|  | p-value | 0.152 | | 0.576 | 0.096 | | | 0.238 | | 0.440 | | 0.875 |
| Δ SBP at 50% Wmax | r | 0.245 | | -0.058 | -0.256 | | | -0.261 | | -0.042 | | -0.110 |
|  | p-value | 0.118 | | 0.715 | 0.102 | | | 0.095 | | 0.791 | | 0.489 |
| Δ DBP at 50% Wmax | r | 0.297 | | 0.023 | **-0.364*** | | | **-0.421**** | | -0.296 | | -0.219 |
|  | p-value | 0.056 | | 0.884 | **0.018** | | | **0.005** | | 0.057 | | 0.163 |
| Δ SBP at 75% Wmax | r | -0.057 | | -0.017 | 0.137 | | | 0.048 | | 0.154 | | -0.051 |
|  | p-value | 0.732 | | 0.919 | 0.412 | | | 0.775 | | 0.357 | | 0.761 |
| Δ DBP at 75% Wmax | r | 0.167 | | -0.156 | -0.008 | | | -0.149 | | -0.151 | | -0.221 |
|  | p-value | 0.316 | | 0.351 | 0.961 | | | 0.373 | | 0.365 | | 0.182 |
| Δ SBP at 100% Wmax | r | 0.085 | | -0.088 | -0.029 | | | -0.059 | | -0.001 | | -0.038 |
|  | p-value | 0.565 | | 0.554 | 0.845 | | | 0.692 | | 0.992 | | 0.797 |
| Δ DBP at 100% Wmax | r | 0.050 | | -0.019 | 0.036 | | | -0.143 | | -0.135 | | 0.058 |
|  | p-value | 0.744 | | 0.899 | 0.812 | | | 0.342 | | 0.373 | | 0.702 |
| Δ SBP at 25W | r | 0.055 | | 0.034 | -0.077 | | | -0.134 | | -0.120 | | -0.018 |
|  | p-value | 0.718 | | 0.825 | 0.613 | | | 0.380 | | 0.430 | | 0.908 |
| Δ DBP at 25W | r | 0.235 | | -0.064 | **-0.360*** | | | -0.189 | | -0.147 | | -0.084 |
|  | p-value | 0.121 | | 0.676 | **0.015** | | | 0.214 | | 0.336 | | 0.582 |
| Δ SBP at 50W | r | 0.029 | | 0.106 | -0.171 | | | -0.096 | | -0.159 | | -0.059 |
|  | p-value | 0.847 | | 0.477 | 0.251 | | | 0.523 | | 0.287 | | 0.693 |
| Δ DBP at 50W | r | 0.231 | | -0.146 | -0.266 | | | -0.122 | | -0.121 | | -0.059 |
|  | p-value | 0.118 | | 0.326 | 0.071 | | | 0.413 | | 0.418 | | 0.692 |
| Δ SBP at 75W | r | 0.025 | | 0.030 | -0.047 | | | -0.076 | | -0.045 | | -0.085 |
|  | p-value | 0.865 | | 0.839 | 0.752 | | | 0.613 | | 0.765 | | 0.572 |
| Δ DBP at 75W | r | 0.215 | | 0.037 | **-0.298*** | | | **-0.368*** | | **-0.316*** | | -0.138 |
|  | p-value | 0.146 | | 0.805 | **0.042** | | | **0.011** | | **0.030** | | 0.355 |
| Δ SBP at 100W | r | 0.015 | | 0.054 | -0.001 | | | -0.122 | | 0.021 | | -0.027 |
|  | p-value | 0.925 | | 0.729 | 0.995 | | | 0.431 | | 0.894 | | 0.860 |
| Δ DBP at 100W | r | 0.157 | | -0.041 | -0.080 | | | -0.267 | | -0.201 | | -0.132 |
|  | p-value | 0.316 | | 0.793 | 0.608 | | | 0.084 | | 0.197 | | 0.397 |
| Δ SBP at 125W | r | -0.040 | | 0.004 | 0.158 | | | -0.034 | | 0.059 | | 0.146 |
|  | p-value | 0.818 | | 0.982 | 0.364 | | | 0.846 | | 0.737 | | 0.403 |
| Δ DBP at 125W | r | 0.290 | | -0.255 | -0.054 | | | -0.246 | | -0.172 | | 0.185 |
|  | p-value | 0.097 | | 0.145 | 0.760 | | | 0.160 | | 0.331 | | 0.295 |
| Δ SBP at 150W | r | 0.444 | | -0.268 | -0.291 | | | -0.272 | | -0.259 | | -0.240 |
|  | p-value | 0.057 | | 0.267 | 0.227 | | | 0.260 | | 0.284 | | 0.323 |
| Δ DBP at 150W | r | 0.125 | | 0.094 | 0.022 | | | -0.420 | | **-0.566*** | | -0.272 |
|  | p-value | 0.610 | | 0.701 | 0.928 | | | 0.073 | | **0.011** | | 0.261 |
| Δ SBP at 175W | r | 0.051 | | **-0.915*** | 0.582 | | | 0.265 | | -0.144 | | -0.189 |
|  | p-value | 0.923 | | **0.011** | 0.226 | | | 0.612 | | 0.785 | | 0.721 |
| Δ DBP at 175W | r | 0.277 | | -0.622 | 0.372 | | | -0.296 | | -0.173 | | -0.350 |
|  | p-value | 0.595 | | 0.187 | 0.468 | | | 0.569 | | 0.743 | | 0.497 |
|  |  |  |  | | |  |  | |  | |  | |

Accelerometry variables were analysed as proportion of daily wear time. SBP = systolic blood pressure, DBP = diastolic blood pressure, % Wmax = percentage of maximal power output, SB = sedentary behavior, LPA = light physical activity, MVPA = moderate-to-vigorous physical activity. *p<0.05. Statistically significant correlations are bolded.
